# ^18^F-NaF PET/CT identifies muscular and subcutaneous calcifications in both dermatomyositis- and systemic sclerosis-related calcinosis

**DOI:** 10.3389/fnume.2025.1593825

**Published:** 2025-05-30

**Authors:** Carrie Richardson, Mehrbod S. Javadi, Ami A. Shah, Caoilfhionn Connolly, Lilja B. Solnes, Fredrick M. Wigley, Laura K. Hummers, Lisa Christopher-Stine

**Affiliations:** ^1^ Division of Rheumatology, Johns Hopkins University, Baltimore, MD, United States; ^2^ Division of Rheumatology, Northwestern University, Chicago, IL, United States; ^3^ Department of Radiology, Johns Hopkins University, Baltimore, MD, United States

**Keywords:** calcinosis, scleroderma (or systemic sclerosis), dermatomyositis, ^18^F-NaF PET/CT, imaging

## Abstract

**Background:**

Calcinosis is a morbid complication of dermatomyositis (DM) and systemic sclerosis (SSc) with no effective pharmacologic treatment or validated whole-body assessment modality. ^18^F-NaF PET/CT may help to quantify and characterize calcinosis.

**Methods:**

In this pilot study, we enrolled three adults with DM and three with SSc, all with new calcinosis deposits. Each underwent ^18^F-NaF PET/CT and clinical examination with semi-quantitative scoring of calcinosis. We described the ^18^F-NaF PET/CT findings and compared these to CT imaging alone as well as to clinical examination.

**Results:**

Calcinosis was noted on ^18^F-NaF PET/CT in the subcutaneous tissue in all patients and the muscle in three patients, including two with SSc. The average semi-quantitative score was 23.5 by ^18^F-NaF PET/CT and 20 by clinical exam. Wilcoxon signed rank test indicated greater scores by ^18^F-NaF PET/CT than by clinical exam (*p* = 0.0264). ^18^F-NaF uptake varied among calcinosis deposits and occurred without corresponding calcifications on CT.

**Conclusions:**

^18^F-NaF PET/CT appears to be a sensitive method of detecting and characterizing calcinosis that provides both quantitative and qualitative data beyond what can be obtained by physical examination or CT alone. ^18^F-NaF uptake occurs in muscle in both SSc and DM, suggesting the possibility that myositis may be driving calcinosis in a subset of patients with SSc.

## Introduction

Calcinosis in dermatomyositis and systemic sclerosis (SSc; scleroderma) is ectopic soft tissue calcification that occurs in the absence of serum abnormalities of calcium and phosphorous. Calcinosis is common in dermatomyositis and SSc, affecting 30%–40% of patients, and causes substantial morbidity (1–4). There is no known effective pharmacologic intervention for calcinosis, and there is no established quantitative measure of whole-body disease burden.

^18^F-NaF PET/CT may be useful for identification, localization, and/or quantification of calcinosis burden or activity in dermatomyositis and SSc (5). ^18^F-NaF is a radiotracer used for detection of hydroxyapatite, which is the principal mineral component of bone as well as of dermatomyositis- and SSc- related calcinosis (6–8). For other disorders involving aberrant ossification or calcification, ^18^F-NaF PET/CT is highly sensitive. For example, in fibrodysplasia ossificans progressiva, which is characterized by heterotopic ossification at sites of trauma, ^18^F-NaF uptake on PET/CT identifies preclinical areas of ossification (9). Furthermore, in atherosclerosis, which is characterized by hydroxyapatite deposition within blood vessels, increased vascular ^18^F-NaF uptake is detectable in areas without radiographic coronary calcium and is an independent predictor of subsequent progressive coronary artery calcification (10, 11). In prostate cancer, ^18^F-NaF PET/CT is 97% sensitive and 94% specific for the detection of bony metastases (12). These studies demonstrate that ^18^F-NaF PET/CT accurately localizes and quantifies ectopic calcification in a variety of contexts and support the use of this imaging modality to assess calcinosis in dermatomyositis and SSc.

Development of effective therapies for calcinosis hinges upon being able to measure calcinosis disease burden. Image-based quantification of calcinosis with ^18^F-NaF PET/CT could provide an objective measure for use in clinical trials as well as a foundation for further mechanistic studies. To this end, we conducted a pilot study to demonstrate the feasibility of using this imaging modality and to compare characteristics of dermatomyositis- and SSc-related calcinosis on ^18^F-NaF PET/CT to expert clinical exam. We hypothesized that there may be areas of ^18^F-NaF uptake on PET without a CT correlate and that ^18^F-NaF PET/CT may be more sensitive than expert exam for the detection of calcinosis.

## Methods

### Patient selection

We recruited patients from the Johns Hopkins Myositis Center and the Johns Hopkins Scleroderma Center, which are both based out of a tertiary medical center in Baltimore, Maryland (USA). We included patients at least 18 years of age with a clinical diagnosis of dermatomyositis or SSc who had at least one new self-reported calcinosis deposit within the 6 months prior to enrollment. We excluded patients who had received more than 2.0 REM of radiation exposure over the previous year, were pregnant or were trying to become pregnant, had recent surgery, or had any metabolic disorder contributing to ectopic calcification. The Institutional Review Board of Johns Hopkins University approved this study, and we obtained written informed consent from all patients.

### Clinical assessment of calcinosis

A single examiner (CR) assessed 26 body areas (bilateral fingers, hands, wrists, forearms, elbows, upper arms, shoulders, thighs, knees, lower legs, ankles, feet as well as head/neck, trunk, pelvic girdle within 1 week of each scan. A board-certified rheumatologist with expertise in the assessment of patients with dermatomyositis (LC-S) or SSc (AAS, LKH, or FMW) verified these examination findings during the same visit. We scored each body area as follows: 0 for no calcinosis, 1 for a <3 small deposits (light burden), or 2 for at least one large (≥3 cm) deposit and/or at least 3 deposits (heavy burden). The expert examiners were masked as to the results of the ^18^F-NaF PET/CET but were familiar with each patient's clinical history.

### ^18^NaF PET/CT imaging protocol

First, we administered 0.15 mCi/kg of ^18^F-NaF, followed by at least 10 ml of saline flush, intravenously to each patient. Second, each patient drank a 12-ounce bottle of water and voided 60 min after infusion of the radiotracer. Third, we positioned each patient on the PET/CT scanner bed for a vertex-to-toes PET/CT protocol in the arms down position. Fourth, we acquired a low dose CT from the vertex to the toes for attenuation correction. Following the CT, we performed a PET scan at 3 min per bed position. We estimated the total amount of radiation exposure at 2.14 REM per patient. All patients tolerated the scan well.

### ^18^F-NaF PET/CT reading

Two board-certified radiologists (MSJ, LBS) read the ^18^F-NaF PET/CT images and arrived at a consensus for each scan. Both readers were masked as to each patient's clinical history as well as to the physical examination scoring. We assessed the presence or absence of ^18^F-NaF uptake in each body area and recorded the maximum standardized uptake value (SUV) for each area with radiotracer uptake. We assessed calcinosis burden on ^18^F-NaF PET/CT for each of the 26 aforementioned body areas and scored each body area on ^18^F-NaF PET/CT as 0 for no calcinosis, 1 for <3 small deposits, and 2 for at least 1 large (≥3 cm) deposit or more than a few small deposits. Readers also commented on qualitative aspects of the scans as appropriate, including precise anatomical locations of deposits, qualitative description of the ^18^F-NaF uptake, and/or whether uptake was associated with CT evidence of calcification.

### Statistical analysis

Comparisons between clinical examination and ^18^F-NaF PET/CET were made using two-tailed Student's *t*-test and Wilcoxon rank sum test as appropriate. A *p* value of <0.05 was considered significant. Statistical analyses were performed using Stata 14.1 (StataCorp, College Station, TX).

## Results

### Patient characteristics

Ages of the patients ranged from 32 to 65 years at the time of the study, and all of the patients were female. The demographic, clinical, and serologic characteristics of the patients are listed in Table 1. Of the three patients with dermatomyositis, one had clinically amyopathic dermatomyositis by modified Sontheimer criteria, and 2 patients with dermatomyositis met Bohan and Peter criteria for probable dermatomyositis (13, 14). All three patients with SSc met 2013 American College of Rheumatology Classification Criteria for SSc (15). None of the patients with SSc had a clinical diagnosis of dermatomyositis overlap. All three patients with SSc had diffuse cutaneous involvement, and all patients with dermatomyositis had characteristic rashes. Patient 1 had received anakinra for calcinosis, most recently 1 year prior to the scan. Patients 3 and 5 had received pamidronate for calcinosis more than 2 years prior to the scan, and patient 4 was receiving treatment with pamidronate for calcinosis at the time of the scan. Patient 2 had received denosumab for osteoporosis within the 6 months prior to the scan. All patients had been treated with immunosuppressive or immunomodulatory therapy in the past, as listed in the table.

**Table 1:** Demographics and disease characteristics of patients.

Patient characteristics	Patient 1	Patient 2	Patient 3	Patient 4	Patient 5	Patient 6
Age, years	65	65	44	64	59	32
Sex	F	F	F	F	F	F
Race	Wh	Wh	Wh	Wh	Wh	Wh
Diagnosis	Diffuse SSc	Diffuse SSc	Diffuse SSc	DM	DM	Amyopathic DM
Disease duration, years	13	12	7	13	21	9
Years since calcinosis onset	9	10	5	6	18	9
Disease manifestations	Diffuse skin thickening, Raynaud's, inflammatory arthritis, sicca, GI dysmotility	Diffuse skin thickening, telangiectases, GI dysmotility (no Raynaud's)	Diffuse skin thickening, inflammatory arthritis, Raynaud's with digital ischemia, GI dysmotility, PAH, ILD	Weakness, rashes	Rashes	Rashes
Autoantibodies	ANA 1:640 speckled, +CCP, +RF, +SSA, +SSB	ANA 1:320 speckled	ANA, +PM/Scl	ANA 1:160 speckled, +NXP2	+p155/140, +PM75	Unidentified antibody
Past Immunomodulatory treatments	MTX, adalimumab, prednisone, anakinra		MTX, HCQ, adalimumab, prednisone	MTX, AZA,	MTX, RTX, HCQ, TAC, cyclosporine, LEF	HCQ
Active immunomodulatory treatments	MTX, adalimumab, prednisone 3 mg daily	Denosumab, prednisone 4 mg daily	MMF 500 mg PO BID	IVIG 2 g/kg Q5 weeks, prednisone 5 mg daily	MMF 1,500 mg PO BID, prednisone 10 mg daily	Prednisone 20 mg, MTX 17.5 mg weekly
Past calcinosis treatments	Anakinra, surgical excision	None	Pamidronate, minocycline, topical sodium thiosulfate, colchicine		Diltiazem, warfarin, dapsone, pamidronate	None
Current calcinosis treatments	None	None	None	Pamidronate	None	None
Osteoporosis or osteopenia	Yes	Yes	Yes	Yes	No	No
Acro-osteolysis	No	No	Yes	No	No	No
History of cancer	No	Basal Cell	No	No	No	No

Wh, white; SSc, scleroderma; DM, dermatomyositis; GI, gastrointestinal; PAH, pulmonary arterial hypertension; ILD, interstitial lung disease; MTX, methotrexate; MMF, mycophenolate mofetil; HCQ, hydroxychloroquine; AZA, azathioprine; IVIG, intravenous immunoglobulin; RTX, rituximab; TAC, tacrolimus; LEF, leflunomide.

### Quantitative comparisons of ^18^F-NaF PET/CT and clinical examination results

^18^F-NaF-PET/CT detected calcinosis in more body areas than clinical exam. Expert exam detected calcinosis clinically in 46.2% (72/156) of all of the body areas examined, compared to (89/156) body areas, or 57.1%, for ^18^F-NaF PET/CT (*p* = 0.0541). Calcinosis was detected more often by ^18^F-NaF PET/CT than clinical exam in the upper arms, feet, shoulder, leg, elbow, and knee. The average semi-quantitative score was 23.5 by ^18^F-NaF PET/CT and 20 by clinical exam. Wilcoxon signed rank test resulted in a *p*-value of 0.0264, indicating greater scores by ^18^F-NaF PET/CT than clinical exam.

### Qualitative ^18^NaF PET/CT scan results

**Figure 1:**
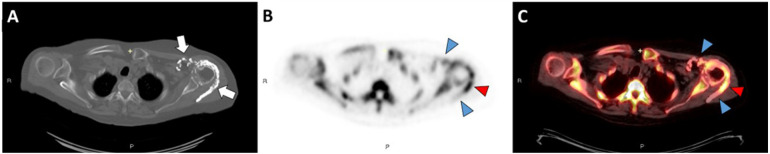
Axial CT image in bone windows **(A)** demonstrates periarticular calcinosis of the left shoulder involving the rotator cuff interposed between the deltoid and infraspinatus muscles (white arrows). Axial ^18^F-NaF PET/CT images **(B,C)** demonstrate intense activity at the mid/distal component of the calcification (red arrowheads) and mild activity along the proximal component and anterior calcifications (blue arrowheads). There is also increased ^18^F-NaF uptake at the left shoulder compared to the right, suggesting arthritic remodeling.

**Figure 2:**
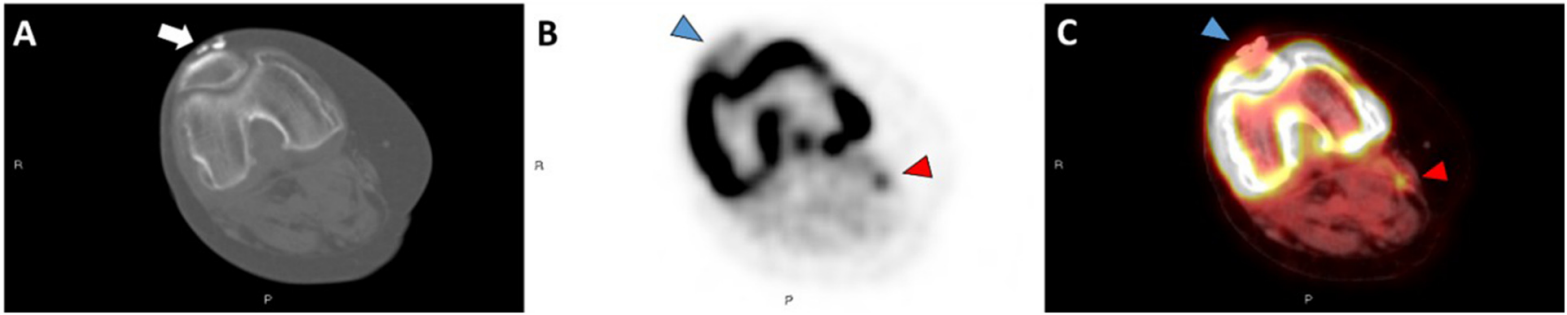
Axial CT image in bone windows **(A)** demonstrates calcinosis involving the quadriceps and patellar tendon complex overlying the left patella in patient 5. Axial ^18^F-NaF PET/CT images **(B,C)** demonstrate mild activity along this calcified complex (blue arrowheads). However, there is focal PET signal activity located along the semimembranosus muscle without associated calcification on CT (red arrowheads).

**Figure 3:**
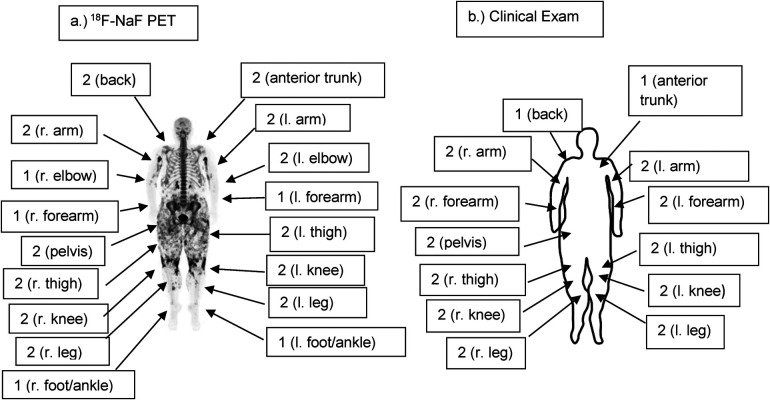
**(a)** Full-body coronal ^18^F-NaF PET imageof patient 6 demonstrating the distribution and intensity of ^18^F-NaF uptake. Note the intense activity in the upper arms and around the knees as well as widespread uptake in the thighs and pelvic girdle. Semi-quantitative radiographic scores are noted, with scores of 0 where not noted (head/neck, shoulders, fingers, hands). **(b)** Semi-quantitative clinical exam scores of Patient 6 are similar to radiographic scores. However, calcinosis was not noted clinically in the feet/ankles or elbows on clinical exam, whereas it was present in these areas radiographically. Clinical scores are 0 where not noted (head/neck, shoulders, fingers, hands, elbows, feet/ankles).

^18^F-NaF PET/CT demonstrated calcinosis in the subcutaneous layer in all 6 patients. However, 1 of 3 DM patient had calcinosis in the muscles as well as subcutaneous tissue, and 2 of 3 SSc patients had calcifications in the muscles as well as the subcutaneous tissue. ^18^F-NaF uptake was variable in areas of calcinosis that appeared uniform on CT. For example, there was intra-lesion variation in ^18^F-NaF uptake in sheets of calcification that appeared uniform on CT in and around the shoulder of Patient 1 (Figure 1). This patient also had calcifications of the rotator cuff as well as variable uptake in the anterior trunk. Patient 5 also had variable uptake in the hip girdle region, where there was intense ^18^F-NaF uptake overall. Patients 2 and 5 had calcification on CT without any ^18^F-NaF uptake on PET. Conversely, Patient 5 also had ^18^F-NaF uptake in the left leg without a CT correlate (Figure 2), and Patient 6 had multiple areas of non-calcified radiotracer uptake along with increased ^18^F-NaF uptake in the axial skeleton, indicative of increased skeletal bone turnover (Figure 3). As illustrated in Figure 3, ^18^F-NaF PET/CT also detected calcinosis in the elbows and foot/ankle regions, which was not detected on clinical exam for this patient.

## Discussion

In this pilot study, we found that ^18^F-NaF PET/CT offered additional quantitative and qualitative data beyond what could be obtained from expert exam or CT alone in dermatomyositis- and SSc-related calcinosis. Consistent with the findings of Atzeni et al. (5), ^18^F-NaF PET/CT identified more calcinosis than appreciated on expert examination, and there was ^18^F-NaF uptake in areas where calcification was not apparent on CT. Based on previous studies demonstrating that ^18^F-NaF uptake predicts the development of ossification in fibrodysplasia ossificans progressiva and the progression of calcification in atherosclerosis, we hypothesize that these areas of ^18^F-NaF uptake may ultimately progress to radiographic calcification. We also found calcification in the muscles in 3 of 6 patients, including 2 with SSc, suggesting that calcifications may originate in the muscular layer due to active myositis. We also found that markedly increased skeletal bone turnover accompanied calcinosis in one patient. In light of the association between calcinosis and osteoporosis (16), this suggests that accelerated bone loss may accompany calcinosis in some patients.

Our study has limitations. Most notably, this was a pilot study with a small sample size of 6 patients, all of whom were white females between the ages of 30 and 65. Because of the small sample size, the power of our study was limited. Notably, our study was not able to detect a statistically significant difference between the number of calcified body areas detected by ^18^F-NaF PET/CT and by expert exam. Future studies would ideally include larger groups of patients to improve power as well as a more diverse patient population to improve generalizability. Furthermore, while there is a validated scoring system for hand calcinosis in systemic sclerosis (17), there is no validated scoring system for full-body calcinosis in either systemic sclerosis or dermatomyositis. Therefore, our study used a semi-quantitative scoring system to assess calcinosis burden as none (0), light (1), or heavy (2) in 26 body areas. Our study demonstrated that this scoring system is feasible in a small cohort, but this scoring system needs to be validated in a future study.

While ^18^F-NaF PET/CT involves greater time, expense, and radiation exposure than other imaging modalities for assessment of calcinosis, its unique characteristics make it a superior imaging modality. In contrast to computed tomography alone, the use of ^18^F-NaF PET/CT provides a functional assessment of hydroxyapatite deposition, as animal studies have demonstrated that ^18^F-NaF uptake is closely related to bone turnover (18). Compared to ^99m^Tc based bone imaging, ^18^F-NaF PET/CT has more robust attenuation correction and scatter correction and higher spatial and contrast resolution (19). Furthermore, ^18^F-NaF PET/CT is amenable to quantitative analysis including kinetic modeling and can provide a quantitative assessment of bone perfusion and bone turnover (20). These characteristics make ^18^F-NaF PET/CT a particularly promising modality for use in clinical trials of calcinosis, as it provides granular, quantitative assessments of whole-body calcinosis and may be able to identify actively forming calcinosis deposits amenable to pharmacologic therapy. Our study demonstrates that ^18^F-NaF PET/CT is well-tolerated by patients and feasible as an assessment modality for calcinosis in future studies. Subsequent longitudinal studies should examine the sensitivity of this imaging modality to changes in calcinosis burden over time as well as whether ^18^F-NaF uptake predicts progression of calcinosis in DM and SSc. Further studies should also assess and validate clinically meaningful thresholds of ^18^F-NaF uptake and whole-body quantitative calcinosis burden on ^18^F-NaF PET/CT, incorporating patient-reported outcome measures.

Calcinosis is a painful, disfiguring and debilitating complication of SSc and DM, and efforts to find an effective therapy have been stymied by a lack of quantitative outcome measures. Our study suggests that ^18^F-NaF PET/CT may have advantages over expert clinical exam or CT alone for detection and quantification of calcinosis. We suggest that ^18^F-NaF PET/CT is a useful imaging modality for identification and quantification of calcinosis activity.

## Data Availability

The datasets presented in this article are not readily available because this dataset involves a small number of participants. The raw dataset will not be made available due to possible breach of privacy associated with transfer of data. Requests to access the datasets should be directed to Lisa Christopher-Stine, lchrist4@jhmi.edu.
